# Skeletal muscle homeostasis and plasticity in youth and ageing: impact of nutrition and exercise

**DOI:** 10.1111/apha.12532

**Published:** 2015-06-21

**Authors:** M. S. Brook, D. J. Wilkinson, B. E. Phillips, J. Perez‐Schindler, A. Philp, K. Smith, P. J. Atherton

**Affiliations:** ^1^ MRC‐ARUK Centre of Excellence for Musculoskeletal Ageing Research, Clinical Metabolic and Molecular PhysiologyUniversity of NottinghamRoyal Derby Hospital CentreDerbyUK; ^2^ MRC‐ARUK Centre of Excellence for Musculoskeletal Ageing Research, School of Sport, Exercise and Rehabilitation SciencesUniversity of BirminghamBirminghamUK

**Keywords:** ageing, muscle, protein turnover, substrate metabolism

## Abstract

Skeletal muscles comprise a substantial portion of whole body mass and are integral for locomotion and metabolic health. Increasing age is associated with declines in both muscle mass and function (e.g. strength‐related performance, power) with declines in muscle function quantitatively outweighing those in muscle volume. The mechanisms behind these declines are multi‐faceted involving both intrinsic age‐related metabolic dysregulation and environmental influences such as nutritional and physical activity. Ageing is associated with a degree of ‘anabolic resistance’ to these key environmental inputs, which likely accelerates the intrinsic processes driving ageing. On this basis, strategies to sensitize and/or promote anabolic responses to nutrition and physical activity are likely to be imperative in alleviating the progression and trajectory of sarcopenia. Both resistance‐ and aerobic‐type exercises are likely to confer functional and health benefits in older age, and a clutch of research suggests that enhancement of anabolic responsiveness to exercise and/or nutrition may be achieved by optimizing modifications of muscle‐loading paradigms (workload, volume, blood flow restriction) or nutritional support (e.g. essential amino acid/leucine) patterns. Nonetheless, more work is needed in which a more holistic view in ageing studies is taken into account. This should include improved characterization of older study recruits, that is physical activity/nutritional behaviours, to limit confounding variables influencing whether findings are attributable to age, or other environmental influences. Nonetheless, on balance, ageing is associated with declines in muscle mass and function and a partially related decline in aerobic capacity. There is also good evidence that metabolic flexibility is impaired in older age.

## Skeletal muscle in health, disease and ageing: an overview

In constituting ~40% of body weight, skeletal muscle is the largest organ in the body, one of the fundamental roles of skeletal muscle is to maintain skeletal structure and locomotion enabling completion of essential daily activities (Reid & Fielding 2012). Skeletal muscle shows a remarkable ability to endure a variety of demands, from producing large feats of strength to sustaining of endurance over long periods of time. Additionally, skeletal muscle possess marked adaptive and regenerative capacity in response to exercise and injury, being able to regenerate even following catastrophic crush (Lepper *et al*. 2011). Skeletal muscles not only permit locomotory activity, but also act as a major control hub over whole‐body metabolic health (Zurlo *et al*. 1990). For instance, skeletal muscle represents the largest site for glucose disposal (Defronzo *et al*. 1985, Shulman *et al*. 1990) and contains large deposits of amino acids (AAs) for liberation in times of stress or fasting [e.g. providing carbon backbones for hepatic gluconeogenesis (Pozefsky *et al*. 1976)]. Therefore, maintenance of skeletal muscle throughout the life course not only preserves physical independence, but also confers protection from a host of metabolic morbidities such as insulin resistance (Rizzoli *et al*. 2013). Typically, muscle mass remains stable during early life; nonetheless, after age ~50 years, muscle mass declines at a rate of ~1% year^−1^ in men and ~0.5% in women (Mitchell *et al*. 2012). This muscle loss may be masked by body weight maintenance via associated accumulation of fat mass (Gallagher *et al*. 2000). Loss of muscle contractile protein material is linked to declines in strength (Frontera *et al*. 1991), mainly due to a decrease in ‘fast’ type II fibre cross‐sectional area (CSA) (Lexell *et al*. 1988, Verdijk *et al*. 2014). Loss of muscle mass is more pronounced in the lower extremities (Janssen *et al*. 2000), an influential factor in age‐related functional impairment (Janssen *et al*. 2002), poor quality‐of‐life (Fielding *et al*. 2011) disability and mortality risk (Metter *et al*. 2002).

Muscle loss with ageing was termed sarcopenia, in part, to promote scientific interest and research in this important area (Rosenberg 1997). Development of sarcopenia is of multi‐factorial consequence, being associated with hormone imbalances (Morley *et al*. 1997, Feldman *et al*. 2002), chronic inflammation (Visser *et al*. 2002, Schaap *et al*. 2009), neurodegeneration (McNeil *et al*. 2005), ectopic fat deposition (Goodpaster *et al*. 2001), decreased satellite cell functionality (Kadi *et al*. 2004a), blunted responses to anabolic stimuli [e.g. nutrition and exercise (Cuthbertson *et al*. 2005, Kumar *et al*. 2009)] and genetic factors (Phillips *et al*. 2013). Moreover, the trajectory of sarcopenia is very likely to be enhanced by certain lifestyle factors, such as age‐related sedentary behaviour patterns (Kortebein *et al*. 2008), nutritional deficiencies (Houston *et al*. 2008, Paddon‐Jones *et al*. 2008) and acute bouts of hospitalization (Ali *et al*. 2008). Pharmacological strategies aimed at mitigating sarcopenia have proved disappointing (Borst 2004) or with major side effects (e.g. prostate and androgenic hormones). Nonetheless, the trialling of selective androgen receptor modulators (SARMS) and antimyostatin therapies [e.g. antibodies or receptor antagonists (Narayanan *et al*. 2008, Dalton *et al*. 2011, Attie *et al*. 2013, Dobs *et al*. 2013)] may hold some promise, subject to effect size and quantitatively beneficial changes in skeletal muscle function occurring alongside those in mass (Dalton *et al*. 2011). Nonetheless, to date, resistance exercise (RE), the act of loading muscle against an external force, remains the most effective intervention for increasing mass, strength and quality in older age (Fiatarone & O'Neill 1994). That said, challenges associated with implementing exercise regimens, at any age, remain.

The mechanisms regulating loss of skeletal muscle mass with age still remain unclear; however, with the shrinkage of any organ (other than necrosis), they must be due to chronic imbalances between protein synthesis (MPS) and protein breakdown (MPB), that is MPB > MPS. In addition, the decline in muscle mass with age, characterized by muscle fibre atrophy [particularly of type II fibres (Lexell *et al*. 1988)] and aspects of neurodegeneration (McNeil *et al*. 2005), has also been examined in the context of muscle satellite cells (SCs). While there is no consensus on whether or not the SC pool size is affected in older age [some reporting declines (Kadi *et al*. 2004a, Verdijk *et al*. 2007) and others not (Roth *et al*. 2000, Dreyer *et al*. 2006)], recent work out of van Loon's laboratory showed reductions in type II fibre area were associated with advancing ageing in humans, consonant to declines in type II fibre satellite cell content (Verdijk *et al*. 2014). Therefore, both muscle protein turnover and SCs are likely to be central factors and as such will be reviewed in the context of ageing and physical activity. A major barrier to studying these processes is that sarcopenia is a slow, incipient process unlike rapid muscle wasting associated with certain disease [e.g. aggressive cachexias (Tisdale 2009, Williams *et al*. 2012), intensive care (Helliwell *et al*. 1998, Reid *et al*. 2004)], such that acute imbalances in metabolic regulation may be hard to identify as they accumulate over years, rather than days or weeks. Therefore, development of novel methods to permit accurate quantitation of muscle tissue protein metabolism has been and will remain central to unravelling the regulation of sarcopenia and the development of effective strategies to mitigate it. Much of this review will focus upon what is known about the regulation of human skeletal muscle metabolism in youth and ageing, although pre‐clinical work is drawn upon where additive or where human data are lacking, inconclusive or open. A significant amount of findings in this area has been ascertained from the use of stable isotope tracers, and so below we will provide brief summary and explanation of past, present and novel methodologies used in this context.

## Stable isotope tracers to quantify muscle protein turnover

Muscle mass is regulated via the maintenance of a dynamic equilibrium between MPS and MPB. Many methods and models using stable isotope tracers have been refined over the years for the measurement of protein metabolism (Wolfe & Chinkes 2005). However, much of what is known about the responses in muscle to ageing, nutrition (and/or exercise) is generally defined using the ‘gold standard’ fractional synthetic rate (FSR) technique. Here, using continuous, bolus or pulsed (or combinations of all three) tracer infusions, rates of incorporation of AA tracer into proteins can be determined, and hence, a FSR calculated using the following equation***:***

$$\text{Fractional Synthesis Rate (FSR)}(\%\cdot h^{-1})=\Delta \text{Em}/\text{Ep}\times 1/t\times 100,$$

where Em (enrichment in muscle) is the change in muscle protein‐bound labelling between two biopsy samples, Ep (precursor enrichment) is the mean labelling over time of the precursor, that is intracellular tRNA (surrogate precursors maybe used such as intracellular, plasma AA or keto acid enrichment), and *t* is the time between biopsies in hours (h). Measurements of FSR are tissue specific and unaffected by blood flow perturbations (unlike A‐V balance techniques), furthermore by simply stopping a steady‐state tracer infusion, the measurement of the decay of the tracer enrichment from the arterial and intracellular pool over time can also give a measurement of fractional breakdown rate (FBR; Zhang *et al*. 1996, 2002). In another approach, using arterial–venous (A‐V) balance kinetics, rates of tissue synthesis and breakdown can be determined by monitoring the rate of disappearance of the tracer from the arterial pool (as a proxy of synthesis), or the rate of appearance of the tracer into the venous pool (as a proxy of breakdown), assuming that the AA being studied is not subject to secondary metabolism within the tissue. While typically restricted to acute study settings (<12 h) (principally due to the need for controlled clinical laboratory settings, intravenous lines and multiple biopsies), the recent reintroduction of the deuterium oxide (D_2_O) tracer and related development of novel methodologies for use in acute (hours–days) and chronic (weeks–months) settings with minimally invasive procedures have been of great interest. For more information on this important technique and its applications to metabolic research, we direct the reader to the following seminal articles (Dufner *et al*. 2005, Robinson *et al*. 2011, MacDonald *et al*. 2013, Wang *et al*. 2014, Wilkinson *et al*. 2014)

## Metabolic and molecular regulation of responses to nutrition

**Figure 1:**
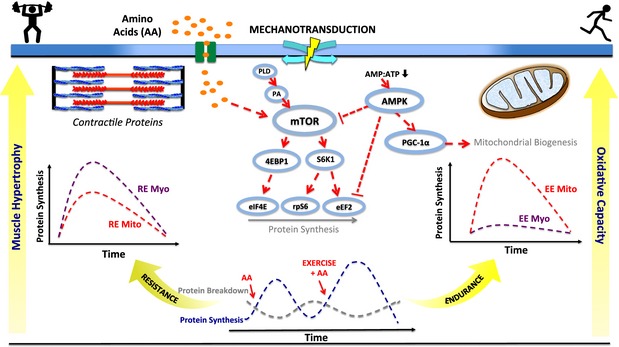
Overview of signalling and muscle proteins synthesis (MPS) responses induced by amino acids (AAs) and different contraction intensities. An increase in intracellular AAs leads to the activation of the mammalian target of rapamycin (mTORC1) and its associated downstream protein substrates: 4E‐binding protein 1 (4E‐BP1) and p70 ribosomal protein S6 kinase 1 (S6K1), promoting assembly of the pre‐initiation complex and mRNA translational efficiency. AA‐induced increases in MPS are transient and return back to baseline despite elevated AAs. Exercise prior to AA availability enhances protein synthetic responses which may persist for >24 h, resulting in greater net protein accretion. Resistance exercise (RE) favours stimulation of myofibrillar (myo) MPS through activation of the mTORC1 pathway, with repeated bouts leading to accumulation of contractile proteins and muscle hypertrophy. Endurance exercise (EE) favours stimulation of mitochondrial (Mito) protein synthesis through activation of 5′ AMP‐activated protein kinase (AMPK) and stimulation of proteins involved in mitochondrial biogenesis. Repeated performance of EE increases muscle mitochondrial content increasing oxidative capacity. Phospholipase D (PLD), phosphatidic acid (PA), adenosine monophosphate (AMP), adenosine triphosphate (ATP), peroxisome proliferator‐activated receptor gamma coactivator 1‐alpha (PGC‐1*α*), eukaryotic translation initiation factor 4E (eIF4E), ribosomal protein S6 (RPS6), eukaryotic elongation factor 2 (eEF2). ↑ represents activation, and Τ represents inhibition.

In humans, rates of MPS in the post‐absorptive state range 0.03–0.07% h^−1^ (Welle *et al*. 1995, Cuthbertson *et al*. 2005, Mittendorfer *et al*. 2005, Kumar *et al*. 2009) and MPB 0.08–0.11% h^−1^ (Phillips *et al*. 1997, 1999), creating an overall negative net balance of −0.01 to −0.08% h^−1^. Therefore, in the post‐absorptive state, rates of MPB > MPS leading to a net negative protein balance and hence a loss of muscle protein. Crucially, this negative protein balance is transiently reversed (MPS > MPB) after food intake (contingent on sufficient high‐quality protein), such that net protein balance is neutral on a daily basis (MPS = MPB). The mechanisms underlying the anabolic effects of food intake involve both the stimulation of MPS (Rennie *et al*. 1982) and suppression of MPB (Wilkes *et al*. 2009). The potent increase in MPS is driven almost entirely by essential amino acids (EAAs) (Smith *et al*. 1992), with the branched chain AA (BCAA: leucine, isoleucine and valine), in particular leucine [and its metabolite(s), e.g. *β*‐hydroxy *β*‐methylbutyric acid (HMB) (Van Koevering & Nissen 1992)] being central to these effects (Wilkinson *et al*. 2013). Although the mechanisms underlying the unique anabolic properties of leucine are incompletely defined, recent work in yeast and cultured mammalians cells has demonstrated that leucyl tRNA synthetase is upstream of activating the hitherto ‘cellular AA sensor’, the mechanistic target of rapamycin complex 1 (mTORC1) in response to leucine (Bonfils *et al*. 2012, Han *et al*. 2012). This was reaffirmed by experiments showing that of all the EAAs, leucine is the most effective EAA in increasing the activity (i.e. phosphorylation) of mTORC1 (Atherton *et al*. 2010b) and its substrates. Indeed, mTORC1 is known to be involved in coordinating MPS responses to nutrition, as anabolic responses to nutrition are ablated when rapamycin, an mTOR inhibitor, is provided alongside EAAs (Dickinson *et al*. 2011). As shown in Figure 1, active mTORC1 stimulates MPS through its substrates: 4E‐binding protein 1 (4E‐BP1) and p70 ribosomal protein S6 kinase 1 (S6K1), promoting assembly of the pre‐initiation complex and mRNA translational efficiency [discussed in Proud (2009, 2014)].

Anabolic responses to nutrient intake are both dose dependent and transient in nature. Maximal increases in MPS are achieved with provision of just 10 g EAA/20 g protein (Cuthbertson *et al*. 2005, Moore *et al*. 2009, Witard *et al*. 2014) with the time course of this response peaking at 1.5–2 h after oral intake, after which there is a decline back to baseline, even in the face of continued availability of EAA substrate (Atherton *et al*. 2010a). This represents the ‘muscle full’ state (Bohé *et al*. 2001) whereby skeletal muscles effectively become anabolically refractory despite enduring EAA availability. While the regulation of this phenomenon is poorly understood, it is speculated that the sensory mechanisms are in some way related to the ensuring complete replacement of muscle protein stores lost during fasted periods; clearly, this refilling ‘set point’ is influenced by physical activity – a notion that will be discussed later on in the review.

In terms of the nutritional regulation of MPB, this has been studied to a far lesser extent, with the temporal profile being as yet undefined. Nonetheless, it has been clearly shown that increases in plasma insulin associated with food intake [i.e. nutrients with pancreatic beta‐cell secretagogue properties (carbohydrates, AAs) (Juntunen *et al*. 2002, Atherton *et al*. 2010a)] are both necessary and sufficient to suppress MPB (Wilkes *et al*. 2009), while raised plasma concentrations of EAAs alone are not (Greenhaff *et al*. 2008). Thus, it is both an increase in MPS and suppression in MPB that govern the characteristic shift from negative‐to‐positive protein balance in postprandial periods.

## Metabolic and molecular regulation of responses to nutrition in older age

Despite these tightly regulated metabolic responses to nutrient intake in healthy younger adults, declines in muscle mass are commonly observed beyond >50 years, even in individuals otherwise considered healthy. This suggests that an alteration in the delicate balance between MPS and MPB is likely to occur in older age. It was initially believed that age‐related reductions in muscle mass were due to significant attenuation in rates of post‐absorptive MPS (Welle *et al*. 1993, 1995, Yarasheski *et al*. 1993, Balagopal *et al*. 1997). This notion has since been largely discredited, with consistent findings from our laboratory (Babraj *et al*. 2005, Cuthbertson *et al*. 2005, Kumar *et al*. 2009, 2012), and many others (Volpi *et al*. 2001, Symons *et al*. 2009, 2011, Markofski *et al*. 2015) revealing equivalent rates of post‐absorptive MPS between healthy younger and older adults. This has led to the seeking of alternative avenues of metabolic investigation that can explain the mechanisms underlying sarcopenia. This led to the theory of anabolic resistance, in which increases in postprandial MPS are less in older compared to younger adults (Volpi *et al*. 2000, Guillet *et al*. 2004, Cuthbertson *et al*. 2005, Smith *et al*. 2012). The thesis being that a repeated inability to recoup post‐absorptive muscle losses during fed periods is driving sarcopenia (Volpi *et al*. 2000, Guillet *et al*. 2004, Cuthbertson *et al*. 2005, Pennings *et al*. 2012). Nonetheless, until recently, direct comparison studies (young vs. old – same nutrition; Table 1) provided mixed results with some reporting equal responses between younger and older participants (Paddon‐Jones *et al*. 2004, Symons *et al*. 2007, 2011) and others revealing a deficit in the responses of older individuals (Cuthbertson *et al*. 2005, Katsanos *et al*. 2005, Smith *et al*. 2012). Nonetheless, a recent study rather definitively showed that higher doses of protein are indeed required for older individuals to generate equivalent anabolic response to younger individuals. This analysis, compiled from a substantial number of younger and older people, definitively demonstrates the existence of anabolic resistance (Moore *et al*. 2014). In terms of MPB, our laboratory reported blunted inhibition of MPB in response to raised plasma insulin concentrations (Wilkes *et al*. 2009). These data highlight insulin resistance of protein metabolism, which likely exacerbates that of resistance to lower doses of protein intake in terms of MPS. Collectively, this desensitization to both of the key anabolic nutrient‐driven stimuli (EAA and insulin) likely promotes sarcopenia.

**Table 1:** The acute effects of nutritional provision on muscle protein turnover in young (Y) and old (O) individuals

Author	Subjects	Route	Feed	FSR period	Muscle fraction	MPS (FSR)	MPB (Ra)	Comment
**Blunted**								
Volpi 2000	4 M 1 F (Y) 4 M 1 F (O)	Oral	40 g CHO+40 g AAs (over 3 h)	0–3 h	Mixed	↑ Y > O	↓ Y = O	MPS increase is impaired in O
Guillet 2004	6 (Y) 8 (O)	IV	Hyperinsulinaemia + Hyperaminoacidaemia	0–4 h	Mixed Mito Sarc	↑ Y > O ↑ Y > O ↑ Y = O		MPS increase is impaired in O
Cuthbertson 2005	20 M (Y) 24 M (O)	Oral	0, 2.5, 5, 10, 20, 40 g EAAs	0–3 h	Myo	↑ Y > O		MPS increases in a dose‐dependent manner saturated at 20 g EAAs, O have lower maximal response
Babraj 2005	8 M (Y) 8 M (O)	Oral	0 or 20 g EAAs (4 Y+O/group)	0–3 h	Myo	↑ Y > O		MPS increase is impaired in O
Katsanos 2005	4 M 4 F (Y) 7 M 4 F (O)	Oral	6.7 g EAAs	0–1, 3.5 h	Phe Kinetics	↑ Y > O (NB)	← →	Phe net uptake 40% greater in Y
Katsanos 2006	8 M 8 F (Y) 12 M 8 F (O)	Oral	(1) 6.7 g EAAs 26% Leu (2) 6.7 g EAAs 41% Leu	0–1, 3.5 h	Mixed	(1) ↑ Y > O (2) ↑ Y > O	(1) ← →(2) ←→	MPS increases only in Y with 27% Leu, Y+O increase MPS with 41% Leu
Smith 2012	8 M 10 W (Y) 10 M 10 W (O)	IV	Hyperinsulinaemia Hyperaminoacidaemia	0–3 h	Mixed	↑ YM >OM ↑ YW >OM		MPS increase is impaired in O
**Not Blunted**								
Volpi 1999	4 M 3 F (Y) 6 M 2 F (O)	Oral	40 g AAs (over 3 h)	0–3 h	Mixed	↑ Y = O	←→	Equal increase in MPS
Paddon‐Jones 2004	2 M 4 F (Y) 3 M 4 F (O)	Oral	15 g EAAs	0–3.5/4 h	Mixed	↑ Y = O	←→	Equal increase in MPS, slower but more sustained positive net balance in O
Symons 2007	5 M 5 F (Y) 5 M 5 F (O)	Oral	113 g Beef	0–5 h	Mixed	↑ Y = O		Equal increase in MPS
Symons 2009	8 M 9 F (Y) 10 M 7 F (O)	Oral	113 g Beef 340 g Beef	0–5 h	Mixed	↑ Y = O ↑ Y = O		Equal increase in MPS, not further enhanced by greater protein
Chevalier 2011	8 W (Y) 8 W (O)	IV	Hyperinsulinaemia + Hyperaminoacidaemia	0–2 h	Mixed	↑ Y = O		Equal increase in MPS

Summary: Provision of sufficient AAs induces a robust increase in MPS in young individuals. Equivalent increases in mixed MPS can be reproduced in old with greater amounts of AA/Leu.

↑, increase; ↓, decrease; ←→, no change; M, male; F, female; IV, intravenous; NB, net balance; EAAs, essential amino acids; AAs, amino acids; CHO, carbohydrate; MPS, muscle protein synthesis; Phe, phenylalanine; FSR, fractional synthetic rate.

If this is to be an important mechanism underlying sarcopenia, then identifying regulatory mechanisms and providing means to mitigate it are clearly central. Attempts have been made to enhance MPS responses to nutrition in older adults, for instance, by both increasing the amount of protein consumed (Symons *et al*. 2009, Pennings *et al*. 2012) and the leucine content (Katsanos *et al*. 2006, Rieu *et al*. 2006, Casperson *et al*. 2012). For example, it has been reported that increasing protein intake from 10 to 35 g in a single bolus leads to a ~50% enhancement of MPS in older men (Pennings *et al*. 2012), while supplementation of the RDA (0.8 g kg^−1^ body weight) of protein with additional leucine over a 2‐week period led to increased rates of MPS in older adults (Casperson *et al*. 2012). Furthermore, 6.7 g of EAAs enriched with leucine enhanced MPS in a group of older adults compared with there being no increase in the absence of supplemental leucine (Katsanos *et al*. 2006); similarly, there was a ~50% increase in MPS in older men who consumed a meal enriched with leucine (Rieu *et al*. 2006). These data highlight that increasing intake of protein/leucine is efficacious for bolstering the anabolic effects of nutrition in older age. To date, there have been no robust investigations into the possible use of insulin sensitizers, or other strategies in relation to the rescuing of insulin‐mediated suppression of MPB (Wilkes *et al*. 2009) in older age.

Neither the mechanisms nor the site(s) of anabolic resistance have been definitively identified. Some have reported reductions in anabolic signalling pathways in muscle, for example mTORC1 and S6K1 phosphorylation (Guillet *et al*. 2004, Cuthbertson *et al*. 2005). Nonetheless, there are multiple level(s) at which this blunting may be regulated – before AAs are transported intracellularly. For example, intestinal absorption, systemic delivery and AA transport (crossing the microcirculation–myocyte interface) can impact upon the efficacy of AAs to act as substrates and/or signals for muscle anabolism (Clark *et al*. 2006). For example, there are reports suggesting systemic availability of AAs is lower in older individuals perhaps as a result of increased first‐pass AA splanchnic extraction (Boirie *et al*. 1997, Volpi *et al*. 1999), that is reduced systemic availability of AAs could indirectly limit muscle anabolism. Nonetheless, under conditions where insulin and glucose are clamped, systemic AA availability is greater in older individuals, possibly accumulating due to lack of uptake in muscle (Cuthbertson *et al*. 2005). Indeed, while trans‐sarcolemmal transport of AAs is rapid and unlikely to be rate limiting (Rennie 1995), recruitment of muscle microvasculature may impact myocyte–AA availability (Clark *et al*. 2000, 2006). Indeed, recruitment of nutritive routes of tortuous capillaries contacting myocytes is essential for muscle perfusion, whereas the non‐nutritive network preferentially supplies muscle connective tissue and adipocytes with minimal myocyte contact (Vincent *et al*. 2005, Sjøberg *et al*. 2011). A number of studies have demonstrated increased microvascular recruitment during feeding. For example, provision of 15 g EAAs resulted in increased microvascular blood flow in young individuals, with early increases in microvascular blood volume followed by later increases in microvascular flow velocity and microvascular blood flow (Mitchell *et al*. 2013). Similarly, provision of AAs and dextrose mirrored responses to a mixed‐meal feed (Vincent *et al*. 2006), in causing enhanced limb blood flow, microvascular blood flow and associated increases in MPS (Phillips *et al*. 2014). This postprandial recruitment of the muscle microvasculature is driven by insulin via mechanisms involving nitric oxide (NO)‐dependent vasodilation of pre‐capillary arterioles (Vincent *et al*. 2004, Rajapakse *et al*. 2013). For example, clamping insulin at 75 *μ*U mL^−1^ led to increased microvascular blood flow (Sjøberg *et al*. 2011), which combined with insulin's known anabolic effects on muscle [suppressing MPB or indirectly stimulating MPS via enhancing delivery of EAAs to the capillary–muscle interface (Wilkes *et al*. 2009)] may prove to be a link between muscle microvascular blood flow and muscle protein anabolism. This supposition is supported by work showing that increases in MPS following femoral artery infusions of various insulin titrations were related to AA delivery via enhancement of microvascular blood flow in an insulin availability‐dependent manner (Timmerman *et al*. 2010a); this suggests that altering the delivery of insulin and EAA to muscle could potentially have profound effects on postprandial muscle anabolism. Yet, interestingly, it has been demonstrated in younger individuals that enhancing limb and microvascular blood flow through intra‐arterial methacholine infusions did not further enhance muscle anabolic responses to feeding (Phillips *et al*. 2014). However, not all data are in agreement with this; work from one group suggests a tight coupling between microvascular blood flow and muscle protein metabolism, demonstrating that the muscle protein anabolic effects of local insulin infusions are inhibited by the NO synthase inhibitor *N* ^G^‐monomethyl‐L‐arginine (L‐NMMA) (Timmerman *et al*. 2010a) and enhanced by the NO donor sodium nitroprusside (SNP) (Timmerman *et al*. 2010b). We suggest that this relationship could in fact be an artefact derived from the use of the A‐V balance 2‐pool method for measures of MPS and MPB, based on an equation using leg blood flow, that is small shifts in tracer concentrations may be amplified by drastically altered blood flow following local insulin or SNP infusions. Finally, in contrast to younger individuals receiving 15 g of EAAs who significantly improved microvascular blood volume, velocity and flow, plus femoral artery blood flow, this response was entirely blunted in older individuals with no increase in macro‐ or microvascular blood flow parameters after feeding (Mitchell *et al*. 2013). Similarly, decrements in limb blood with advancing age were reported with older (vs. younger) individuals exhibiting 20–30% reductions in limb blood flow under post‐absorptive and postprandial conditions (Skilton *et al*. 2005). Nonetheless, recent work from our laboratory showed that exposing older individuals to a RE training programme markedly enhanced microvascular responses to feeding without improving fed‐state MPS (Phillips *et al*. 2015). As such, the site(s) of anabolic resistance very much remain an area that is poorly understood.

## Metabolic and molecular regulation of adaptation to exercise

### Resistance‐type exercise

The literature is awash with studies investigating the effects of RE on muscle protein turnover. This is mainly due to the will to define optimal muscle growth regimens and determine the mechanisms regulating hypertrophy [especially given the significant potential of RE as a non‐pharmacological approach to combating muscle wasting (Liu & Latham 2009, Aagaard *et al*. 2010, Walker *et al*. 2011)]. Similar to that of feeding, RE has been shown to induce a 2‐ to threefold increase in MPS following a single bout of RE (Phillips *et al*. 1997, Kumar *et al*. 2009, Holm *et al*. 2010). Moreover, the anabolic effect of RE is augmented by consumption of protein alongside RE (West *et al*. 2011) such that with adequate nutrition, increases in MPS can be sustained for >24 h (Phillips *et al*. 1997, Miller *et al*. 2005, Cuthbertson *et al*. 2006). This is driven by intake of EAAs extending the duration, rather than amplitude, of muscle anabolic responses to RE. As a result of the potentiation of MPS by EAA intake following RE, net protein balance remains positive despite concordant increases in MPB (Phillips *et al*. 1997, 1999). It is the cumulative effect of repeated bouts of exercise and feeding combinations which drives RE‐induced hypertrophy (Volek *et al*. 2013). As one might expect, there is also a dose–response relationship between RE and MPS: MPS peaks ~1–2 h after RE in the fasted state [abating after 4 h in the absence of EAA intake (Kumar *et al*. 2009, 2012)] and follows a dose‐dependent increase in MPS being near maximal at 60–90% of an individuals 1 RM, when external work is matched between loads for total volume (Kumar *et al*. 2009). Nonetheless, it is not always the case that heavier weights promote greater protein accretion and bigger muscles. This was elegantly highlighted by Burd *et al*. (2010b), who employing a unilateral exercise model, exposed volunteers to RE at 90% 1 RM to fatigue, 30% 1 RM work‐matched to 90% 1 RM group and at 30% 1 RM to fatigue. They found MPS was similarly increased in both 90% 1 RM and 30% 1 RM groups 4 h post‐RE despite major discrepancies in absolute load (Burd *et al*. 2010a,b). However, this effect was only observed when RE was performed to volitional failure at 30% and not when work matched (Burd *et al*. 2010b). This was likely due to increased type II muscle fibre recruitment through fatiguing contractions resulting in maximal fibre recruitment (Burd *et al*. 2010b). In support of this thesis, detailed follow‐up work by the same group showed heightened post‐exercise fed‐state MPS at 30% 1 RM to failure, when the time under tension was increased to 6 s from 1 s (Burd *et al*. 2012); this highlights the importance of classical fibre recruitment paradigms for hypertrophy.

The cellular processes regulating anabolic responses to exercise are more complex than those with nutrition alone as RE triggers multiple intramuscular signalling networks associated with cellular biochemical, mechanical and metabolic stress. Nonetheless, as with MPS responses to nutrition mTORC1 plays a key role in coordinating these responses (Drummond *et al*. 2009), with well‐defined ‘downstream’, mTORC1 substrates consistently upregulated in the hours after RE (Cuthbertson *et al*. 2005, Glover *et al*. 2008a, Kumar *et al*. 2009, Burd *et al*. 2010a, Lundberg *et al*. 2012, Fernandez‐Gonzalo *et al*. 2013). The regulation of mTORC1 by mechanotransduction is yet to be determined; while some authors retain that a canonical pathway of regulation for mTORC1 via IGF1‐PI3K‐Akt/PKB‐mTOR exists, recent evidence has pointed to the existence of muscle intrinsic mechanosensitive signalling pathways, for example through production of the lipid second messenger, phosphatidic acid (PA)/phospholipase D (PLD) (Hornberger *et al*. 2006, O'Neil *et al*. 2009) and adhesome proteins such as focal adhesion kinase (FAK) (Klossner *et al*. 2009) as signalling to activate mTORC1 post‐RE.

Satellite cells (SCs) also play an important role in responses to RE. The physiological role of SCs is to provide nuclei to existing myofibres thereby enabling maintained/enhanced transcriptional capacity, while at the same time ensuring, through self‐renewal, maintenance of the endogenous SC population (Olguin & Olwin 2004, Troy *et al*. 2012). This is achieved through activation of mitotically quiescent SCs upon which asymmetric cell division occurs and one daughter cell is committed to differentiation, while the second becomes quiescent or continues to proliferate (Moss & Leblond 1971). While the role of SCs in mediating repair from crush injury or mycotoxin exposure is established (Carlson 1968, Lefaucheur & Sebille 1995, Lepper *et al*. 2011), the true physiological role of SCs in mediating adaptations to exercise arguably remains a more contentious issue. The most commonly studied aspect of SCs in terms of adaptation to exercise is in the context of muscle hypertrophy (Kadi *et al*. 2004b, Petrella *et al*. 2008). The purported role of SCs in the regulation of hypertrophy was derived from the concept that each nucleus can manage only a certain volume of cytoplasm and that this so‐called karyoplasmatic ratio needs to be maintained (Allen *et al*. 1999). Theoretically, it is argued that as a muscle cell grows, the nucleus content of these terminally differentiated myofibres becomes diluted to a point a new source of nuclei is needed to overcome a ‘ceiling effect’ in growth (Petrella *et al*. 2006, 2008). Thus, the potential importance of SCs in mediating hypertrophy has foundations that warrant discussion. Consistent with a role for SCs in hypertrophy, the recruitment of new nuclei from SC fusing with the pre‐existing muscle fibre syncytia has been noted as a feature of hypertrophy in humans (Kadi *et al*. 1999). Similarly, altered regulation of myogenic regulatory factors (MRFs involved in the activation and proliferation of previously quiescent SCs) is observed in the hours following RE (McKay *et al*. 2008). Further supporting a role for SCs are reports describing the stimulatory effects of short‐ and long‐term RE training programmes upon SC content (Kadi *et al*. 1999, 2004b, Crameri *et al*. 2004, O'Reilly *et al*. 2008). Using a novel approach, Petrella *et al*. (2008) applied cluster analyses to investigate relationships between the degree of hypertrophic responsiveness to RE and SC activity. Using the power of biological variation, they showed that ‘high responders’ for hypertrophy exhibited increased SC number pre‐training and greater myonuclei numbers following resistance training. As high responders expanded their myonuclear domains, the authors suggested this was the driving force behind demand for myonuclear addition from SC sources to support hypertrophy in successful growth adaptors. Nonetheless, it could be argued poor intrinsic capacity for increasing MPS could be the driving force behind the lack of increase in myofibre nuclear number such that the ability to sustain positive increases in net protein balance rather than stimulate SCs was the physiological rate limiting step for hypertrophy. In contrast, data from others have pointed to a poor correlation between fibre CSA and myonuclei number (Bruusgaard *et al*. 2012).

### Aerobic‐type exercise

The major adaptation associated with aerobic‐type exercise (AE) is that of increased capacity for oxygen extraction and utilization (Jones & Carter 2000) principally governed by mitochondrial capacity and function. The relatively small amount of work (vs. RE), which has investigated aerobic exercise (AE) responses, suggests post‐exercise stimulation of mixed muscle MPS (Harber *et al*. 2009a, 2010) is predominantly driven by increases in sarcoplasmic and mitochondrial (Wilkinson *et al*. 2008), rather than myofibrillar MPS (Fig. 1) with AE‐induced increases in mitochondrial synthesis being in evidence 24 h post‐exercise (Di Donato *et al*. 2014). The molecular governance of such selectivity over exercise‐specific synthesis of muscle fractions (i.e. RE: myofibrillar and AE: mitochondrial) remains undefined but is likely governed by non‐stochastic mechanisms, for example the prevailing transcriptional background [which somewhat differs between RE and AE (Coffey *et al*. 2006, Wilkinson *et al*. 2008)]. Similar to RE, the mechanisms regulating induction of mitochondrial MPS are complex. Following initiation of AE, there is a rapid, transient flux of numerous substrates, metabolites and nucleotides within skeletal muscle (Richter *et al*. 1992), factors which are thought to trigger an increase in transcriptional pathways and signal transduction cascades, which ultimately regulate mitochondrial biogenesis programmes. For example, 5′ AMP‐activated protein kinase/p38/protein kinase A (AMPK/p38/PKA) pathway activation induces the upregulation of nuclear and mitochondrial transcription factors such as nuclear receptor 1 (NRF1) and 2 (NRF2), mitochondrial transcription factor A (TFAM) and PGC‐1*α*, all of which modulate mitochondrial biogenesis (Scarpulla 2008). On face value, a central role for PGC‐1*α* in mitochondrial metabolism has been proposed following experiments using transgenic mice with muscle‐specific overexpression of PGC‐1*α* exhibiting enhanced exercise performance, VO_2peak_ and angiogenesis (Calvo *et al*. 2008), and correlative studies in humans. Nonetheless, there is strong evidence that PGC‐1*α* is not required for mitochondrial adaptation to exercise because AE training in whole‐body PGC‐1*α* knockout mice (Leick *et al*. 2008) and muscle‐specific knockout mice (Rowe *et al*. 2012) results in ‘normal’ mitochondrial biogenesis, indicating that substantial redundancy exists in the mitochondrial transcriptional response in skeletal muscle, that is that there are no single master regulators. By extension, transcriptional networking of the adaptive response of human skeletal muscle to AE does not appear to be dependent on PGC‐1*α* signalling (Timmons 2011), further driving home this assertion.

## Metabolic and molecular regulation of adaptation to exercise in older age

### Resistance‐type exercise

Despite extensive investment in pharmaceutical interventions (Onder *et al*. 2009) and the discovery of a number of potential novel targeted pharmaconutrients [ursolic acid (UA), HMB, PA, etc. (Vukovich *et al*. 2001, Kunkel *et al*. 2011, Hoffman *et al*. 2012)], RE with appropriate supportive nutrition remains the current most effective and safe means by which to maintain or increase muscle mass in older adults (Ivey *et al*. 2000, Parise & Yarasheski 2000, Häkkinen *et al*. 2001, Kumar *et al*. 2012). Yet, despite RE being a potent anabolic stimulus, as observed with nutrition, ageing is also associated with blunted MPS responses to RE across a range of exercise intensities (e.g. 30–90% 1 RM) (Sheffield‐Moore 2005, Kumar *et al*. 2009, 2012, Fry *et al*. 2011). This age‐related blunting of acute anabolism is accompanied by concomitant depressions in mTORC1 activation and its associated downstream proteins (Kumar *et al*. 2009, Fry *et al*. 2011) with blunted activation of P70S6K1 and 4EBP1 being demonstrated from 1 h after exercise (Kumar *et al*. 2009) up to 24 h (Fry *et al*. 2011) in older individuals. Similarly, only younger individuals have shown correlations between the extent of mTORC1 and P70S6k1 phosphorylation with MPS (Kumar *et al*. 2009, Fry *et al*. 2011), highlighting their roles in driving MPS and dysregulation with age. Nonetheless, blunted anabolic signalling is not shown by all (Drummond *et al*. 2008, Mayhew *et al*. 2009), although this may simply be a matter of timing. Indeed, not all studies show such a blunting. Although some studies have not directly compared younger and older subjects (Dreyer *et al*. 2008, Yang *et al*. 2012, Churchward‐Venne *et al*. 2014, Dickinson *et al*. 2014, Witard *et al*. 2014), those which have (Table 2), equal results may reflect the fact that the mixed muscle is being primarily measured (Yarasheski *et al*. 1993, Drummond *et al*. 2008, Symons *et al*. 2011), whereas those where myofibrillar components are measured consistently show blunting (Kumar *et al*. 2009, Moore *et al*. 2014). To exemplify the importance of this, despite 60% of mixed muscle consisting of myofibrillar protein, the remaining fraction is a mix of protein turning over at varied rates, which may compound the data showing no difference when this is measured. Irrespectively, there is a clear need to define optimal ways to promote anabolism in older age, using exercise and exercise/nutritional/pharmacological combinations. Recent data from our laboratory suggest that ageing muscle is associated with a shift in sensitivity, meaning that it may take a greater amount of exercise stimulus to ensure a maximal anabolic response, similar to what has been recently reported with nutrition (Moore *et al*. 2014). For example, we observed that older adults who performed three sets of 14 reps unilateral leg extension at 40% 1 RM showed no increase in fasted myofibrillar FSR in the 4 h post‐exercise (Kumar *et al*. 2012). Yet, when the volume of the exercise was increased to six sets of 14 reps with load remaining constant (40% 1 RM), a doubling of FSR was seen 1–2 h post‐exercise, matched with stimulation of P70S6k1 only seen with greater volume (Kumar *et al*. 2012). This reflects the findings of Burd *et al*. (2010a,b) who suggested that maximal stimulation could be achieved at low loads if the volume of work is sufficient for maximal fibre recruitment. This work by Kumar *et al*. (2012) suggests that despite blunted responses to exercise stimuli with ageing, it may be possible to overcome some of this blunting by introducing low‐load high‐volume exercise. This combined with adequate protein intake could assist in slowing the age‐related decline in muscle mass. Finally, given the potent effects of nutrition upon promoting sustainment of anabolic responses to RE, it has been tested whether this blunting may be ‘overcome’ by dietary manipulation. In this context, similar to findings reported at rest (Katsanos *et al*. 2006), enrichment of low‐dose EAAs with the addition of leucine (Churchward‐Venne *et al*. 2012) after RE has shown anabolic efficacy.

**Table 2:** The acute response of muscle protein turnover to exercise in young (Y) and old (O) individuals

Author	Subjects	Exercise	Feed	FSR Period	Muscle fraction	MPS (FSR)	MPB (Ra)	Comment
**Blunted**								
Sheffield‐Moore 2004	6 M (Y) 6 M (O)	45 min walking 40% Vo_2peak_	–	0–10 min, 1 h, 3 h	Mixed	↑ Y > O	↑ Y = O (0–10 min)	Y + O increase MPS 0–10 min but only remains elevated 10–60 min in Y
Sheffield‐Moore 2005	6 M (Y) 6 M (O)	KE 6 × 8, 80% 1 RM	–	0–10 min, 1 h, 3 h	Mixed	↑ Y > O	↑ O (0–10 min)	O increase MPS early 0–10 min declining thereafter. Y show late increase in MPS although sustained for longer, 1 h‐3 h
Kumar 2009	25 M (Y) 25 M (O)	KE 20–90% 1 RM	–	0–1, 2, 4 h	Myo	↑ Y > O		Sigmoidal dose‐related effect of RE, maximized 60–90%. Overall (AUC) Y show 30% >MPS
Mayhew 2009	8 M (Y) 6 M (O)	3 × 8–12 RM KE + LP + Squat	–	~21–24 h	Mixed	↑ Y > O		MPS is only increased in Y, although does not impact eventual muscle gains in O
Fry 2011	8 M 8 W (Y) 8 M 8 W (O)	KE 8 × 10 70% 1 RM	–	0–3, 6 h, 22–24 h	Mixed	↑ Y > O		MPS increase is impaired in O
**Not blunted**								
Drummond 2008	7 M (Y) 6 M (O)	8 × 10 KE 70% 1 RM	20 g EAAs at 60 min	0–1, 3, 6 h	Mixed	↑ Y = O		MPS increase is delayed in O, although equal over 5 h (AUC)
Durham 2010	9 M (Y) 8 M (O)	45 min walking 40% Vo_2peak_	AA infusion	0–10 min, 3 h	Mixed	↑ Y = O	↓ Y = O	Equal increase in MPS, although O show blunted synthetic efficiency
Symons 2011	7 M (Y) 7 M (O)	6 × 8 80% 1 RM KE (60 min post‐feed)	340 g beef	0–5 h	Mixed	↑ Y = O		Equal increase in MPS
Kumar 2012	12 M (Y) 12 M (O)	(1) KE 3 or 6 × 14 40% 1 RM (2) KE 3 or 6 × 8 75% 1 RM	–	0–1, 2, 4 h	Myo	(1) 3 sets ↑ Y > O 6 sets ↑ Y = O (2) 3 sets ↑ Y = O 6 sets ↑ Y = O		Increasing volume enhances post‐exercise response in O (AUC)

Summary: MPS is robustly stimulated in response to resistance exercise in young; however, at the same volume and load, MPS is blunted in older muscle. MPS is seemingly stimulated to similar levels as in young, when a greater volume of exercise is performed in older adults.

↑, increase; ↓, decrease; ←→, no change; M, male; KE, knee extension; LP, leg press; Iso, isokinetic; AUC, area under curve; Myo, myofibrillar; 1 RM, one repetition maximum; EAAs, essential amino acids; AAs, amino acids; CSA, cross‐sectional area; FSR, fractional synthetic rate.

### Aerobic‐type exercise

Rooyackers *et al*. (1996) were the first to report that basal mitochondrial fractional synthesis rates were reduced with ageing. Rooyackers examined basal mitochondrial fractional synthesis rates in younger, middle‐aged and older individuals using a primed continuous infusion of L‐[1^13^C] leucine. The middle‐aged group displayed a 40% reduction in mitochondrial FSR compared to younger individuals, without further declines in the older group (Rooyackers *et al*. 1996). In comparison, mitochondrial enzyme activity (cytochrome c oxidase, citrate synthase) paralleled the younger and middle‐aged FSR showing an age‐associated decline. However, in contrast to the FSR data, mitochondrial enzyme activity continued to decline in the older group, compared to middle‐aged. Thus, it would appear that a reduction in protein synthesis could not fully explain the age‐associated decline in mitochondrial function (Rooyackers *et al*. 1996). Given that protein turnover represents the net balance of synthesis and breakdown, it was suggested that age‐associated modulation of mitochondrial breakdown could also play a role in reductions in mitochondrial content/function (Rooyackers *et al*. 1996). This blunting/reduction in mitochondrial MPS with age is further reflected through a blunted anabolic response to endurance‐type exercise where mixed muscle synthesis rates were reduced in older compared to younger groups following 45 min low/moderate intensity walking (Sheffield‐Moore *et al*. 2004, Durham *et al*. 2010), a blunting which could be primarily driven through a reduced mitochondrial MPS. This data mirrored the anabolic resistance following amino acid ingestion and resistance‐type exercise previously reported (Cuthbertson *et al*. 2005, Kumar *et al*. 2009), suggesting that older muscles have a blunted anabolic response to both aerobic‐ and resistance‐type exercise. Post‐exercise induction of skeletal muscle PGC‐1*α* and mitochondrial biogenesis transcriptional responses to acute AE appears to be maintained with ageing (Cobley *et al*. 2012), suggesting altered activity of other aspects of mitochondrial regulation may be responsible for the reduction in skeletal muscle mitochondrial content/biogenesis with age (Johnson *et al*. 2013).

## Chronic adaptations to exercise in youth and ageing

### Resistance‐type exercise

Repeated bouts of RE lead to a chronic scenario of positive MPS balance (Wilkinson *et al*. 2014) ultimately conferring accumulation of contractile material – the resulting physiological hallmark of which is hypertrophy. With long‐term progressive RE training (RET), increases in fibre dimensions (and unlikely myocellular hyperplasia) culminate in increases in whole muscle CSA (Narici *et al*. 1996, Ahtiainen *et al*. 2003, Hulmi *et al*. 2009). These changes may become apparent after just a few weeks of training (Blazevich *et al*. 2007, Seynnes *et al*. 2007, Norrbrand *et al*. 2008) with gains generally decreasing as training progresses (Wernbom *et al*. 2007). As expected, hypertrophy produced through loading is associated with concomitant improvements in strength, with greater amounts of contractile material able to produce greater force (Garfinkel & Cafarelli 1992, Narici *et al*. 1996). Nonetheless, increases in strength are often reported to be proportionally greater than mass (Jones & Rutherford 1987), which may be in part due to increased neural recruitment activating a larger proportion of the muscle (Häkkinen & Komi 1982). Changes in muscle architecture [pennation angle and fascicle length (Seynnes *et al*. 2007, Franchi *et al*. 2014)] also contribute to muscle hypertrophy and functional improvements (Aagaard *et al*. 2001). Adaptations to RE are influenced by the RE regime and nutritional sufficiency (Campos *et al*. 2002); although for protein intake, the supplemental effect size is rather small (Cermak *et al*. 2012). A major influence in prevailing muscle hypertrophy is one outwith of an individual's control as RE does not result in uniform growth responses between individuals, with a vast range of values reported, that is many showing zero hypertrophy responses to RET. Therefore, hypertrophic adaptation is impacted by individual genetics (Phillips *et al*. 2013); intriguingly, the molecular governance of this remains completely undefined.

Nonetheless, the acid test as to whether short‐term blunted anabolic responses to RE are reflected in the capacity to synthesize muscle is to directly compare younger and older individuals undertaking the same RE regime (Table 3), with acute anabolic differences reflecting the hypertrophic response. In agreement with this, some studies have indicated that whole‐body RET induces greater gains in lean mass (Lemmer *et al*. 2000, Phillips *et al*. 2012) and CSA (Welle *et al*. 1996) in younger than older individuals. Yet, other studies focusing on quadriceps muscles are of mixed consensus with some reporting greater increases in younger (Raue *et al*. 2009, Greig *et al*. 2011), and others equal (Ivey *et al*. 2000, Häkkinen *et al*. 2001, Mayhew *et al*. 2009). At the level of fibre CSA, the available data generally show younger subjects exhibit greater increases in type I fibre CSA (Kosek *et al*. 2006, Martel *et al*. 2006, Mero *et al*. 2013) with increases in type II CSA also being greater (Kosek *et al*. 2006, Raue *et al*. 2009, Mero *et al*. 2013) or equal (Hakkinen *et al*. 1998, Martel *et al*. 2006, Mayhew *et al*. 2009). These discrepancies are likely to arise from variances in training regimes, nutritional support and analytical techniques. Relative progression of training loads may be similar between younger and older individuals (Ivey *et al*. 2000, Kosek *et al*. 2006, Mayhew *et al*. 2009) in part may be due to neural contributions (Hakkinen *et al*. 1998) as equal strength gains have been produced with limited gains in contractile mass in older age (Moritani & deVries 1980, Kosek *et al*. 2006, Mero *et al*. 2013). RE certainly improves muscle function in older age (Macaluso *et al*. 2004, Peterson *et al*. 2011), yet the gains in mass and strength appear to diminish compared with those of younger subjects. Previous studies have highlighted blunted responses in acute MPS and anabolic signalling (Kumar *et al*. 2009, Fry *et al*. 2011), yet as to why a blunted response is produced is still to be unravelled. In terms of the potential role of SCs in maladaptation to RE training, it has been shown that MRFs involved in SC proliferation and differentiation have also shown altered regulation after RE in older individuals (Snijders *et al*. 2014), possibly contributing to the attenuated hypertrophic response (Petrella *et al*. 2006). Although the physiological role of SCs in muscle hypertrophy in ageing remains contentious (Petrella *et al*. 2006, Mackey *et al*. 2007, Verdijk *et al*. 2009), it was recently reported that 3 months of RE training was able to normalize (vs. youthful muscle) SC number in older subjects and with this reverse type II fibre atrophy (Kosek *et al*. 2006). These data that support a tight coupling between SC content and type II fibre size are important data because type II fibres are most amenable to exercise‐induced hypertrophy. Nonetheless, whether this is cause–effect relationship, that is if fibre atrophy/hypertrophy is driving SC depletion/repletion or *vice versa*, remains to be established. Moreover, whether older SCs are subject to apoptosis (Jejurikar *et al*. 2006, Fulle *et al*. 2013), impaired self‐renewal and/or activation in response to physiological cues *in vivo* (Conboy *et al*. 2003, Dreyer *et al*. 2006) and *in vitro* (Schultz & Lipton 1982, Lorenzon *et al*. 2004, Mouly *et al*. 2005) also remains the subject of debate. While there has been no attempt to determine the mechanistic basis of this accounting both for muscle protein turnover and SC activity concomitantly, like with MPS, there have been reports of blunted myogenic responses, indicative of impaired SC activation responses to RE in older age (Snijders *et al*. 2014). Thus, a desensitization to both myogenic and anabolic cues could underpin the apparent blunted responsiveness of older aged skeletal muscles to exercise‐mediated hypertrophic stimuli; clearly, the integrated molecular basis of this is a focus of pursuit. Given evidence that anabolic resistance may be mitigated according to loading and nutritional paradigms, it is perhaps unsurprising that some studies report equal adaptation and others not, especially with lack of data in subject diet and habitual activity. Nonetheless, exposed to the same suboptimal stimulus, muscles of older individuals apparently exhibit desensitized hypertrophic responses.

**Table 3:** Adaptations to resistance exercise in young (Y) and old (O) individuals

Reference	Subjects	Exercise	Volume Sets × Reps (Intensity)	Frequency	1 RM	MVC	Mass changes	
**Blunted**								
Moritani 1980	5 M (Y) 5 M (O)	Elbow flexors	2 × 10 (66% 1 RM)	3 days week^−1^ 12 weeks	↑ Y 29% = O 21%		CSA Skin fold	↑ 5.4 cm Y
Well 1996	5 M 4 F (Y) 4 M 4 F (O)	Whole body	3 × 8 (80% 3 RM) 4 total exercises	3 days week^−1^ 12 weeks	↑ EF 21% Y **> **19% O ↑ KF 38% Y **> **32% O ↑ KE 28% Y = 64% O	–	CSA (MRI)	↑ EF 22% Y > 9% O ↑ KF 8% Y > 2% O ↑ KE 4% Y = 6% O
Lemmer 2001	10 M 10 F (Y) 11 M 11 F (O	Whole body	1–2 × 15 (max contractions) × 6–10 total exercises	3 days week^−1^ 24 weeks	↑ LP 31% Y **> **21% O ↑ CP 28% Y **> **16% O		FFM (DXA)	↑ 2 kg Y >1 kg O
Kosek 2005	13 M 11 F (Y) 12 M 13 F (O)	Lower body	3 × 8–12 (80% 1 RM) ×3 total exercises	3 days week^−1^ 16 weeks	↑ 28–47% Y = 33–39% O	**–**	CSA (Fibre)	↑ Type I CSA Y 18% ↑ Type II Y 32% >23% O
Martel 2006	13 M 9 F (Y) 11 M 7 F (O)	Unilateral KE	5 sets (in total 50 near max contractions)	3 days week^−1^ 9 weeks	↑ 34% Y **> **28% O	**–**	CSA (Fibre)	↑ Type I Y 20% ↑ Type IIa Y 20% = O(M) 24% ↑ Type IIX Y(M) 41% O(F) 49%
Raue 2009	9 W (Y) 6 W (O)	KE	3 × 10 (75% 1 RM)	3 days week^−1^ 12 weeks	↑ 36% Y = 26% O	**–**	CSA (Fibre) CSA (MRI)	↑ Type IIa Y 28% ↑ **Y** 5%
Phillips 2011	9 M 5 F (Y) 10 M 10 F (Mid) 10 M 7 F (O)	Whole body	2 × 12 (70% 1 RM) ×8 total exercises	3 days week^−1^ 20 weeks	↑ 36% Y = 35% Mid = 39% O	**–**	FFM (DXA)	↑ **Y** 5% Mid 1%
Greig 2011	16 W (Y) 9 W (O)	Isometric KE	4 × 15 maximal contractions	3 days week^−1^ 12 weeks		↑ Y 27% >O 16%	Volume (MRI)	↑ 6.2% Y > 2.5% O
Mero 2013	21 M (Y) 18 M (O)	Whole body	2–4 × 5–15 (40–80% 1 RM) ×~10 total exercises	2 days week^−1^ 21 weeks	↑ Y = O	↑ Y = O	Fibre CSA	↑ Type I Y ↑ Type II Y
Mitchell 2015	16 M (Y) 16 M (O)	Whole body (1 upper/2 lower week^−1^)	3–4 (75–85% 1 RM) ×4–6 total exercises	3 days week^−1^ 12 weeks	↑ Y = O	↑ Y = O	Fibre CSA	↑ Type I Y = O ↑ Type II Y **> **O
**Not blunted**								
Hakkinen 1998	8 M (Y) 10 M (O)	Whole body	3–6 × 3–5/6–8/8–10 RM ×7 total exercises	3 days week^−1^ 10 weeks	–	↑ Y 15% = O 16%	CSA (MRI) CSA (Fibre)	↑ QF 12.2% Y = 8.5% O ↑ Type I CSA 22% Y = 23% O ↑ Type II CSA 25% Y = 37% O
Ivey 2000	11 M 9 F (Y) 11 M 11 F (O)	Unilateral KE	5 sets (in total 50 near max contractions)	3 days week^−1^ 9 weeks	↑ Y M 27% F 40% = ↑ O M 26% F 28%	**–**	Volume (MRI)	↑ 12% Y M 5% F = ↑ 11% O M 12% F
Mayhew 2009	21 (Y) 15 (O)	Lower body	3 × 8–12 RM × 3 exercises	3 days week^−1^ 16 weeks	↑ Y 44% = O 38%	**–**	CSA (Fibre) FFM (DXA)	↑ Type IIa Y 37% = O 42% ↑ Y 5% = O 6%

Summary: Resistance exercise has benefits on muscle mass and strength in both Y and O, yet Y individuals show consistently a hypertrophic responses.

↑, increase; M, male; KE, knee extension; KF, knee flexion; LP, leg press; CP, chest press; EF, elbow flexion; CSA, cross‐sectional area.

### Aerobic‐type exercise

The use of aerobic exercise as an intervention against sarcopenia has been less explored, due to the lack of perceived increases in mass and strength. Nonetheless, with long‐term training, AE can increase muscle mass and function with age (Harber *et al*. 2009b) and have shown increases in mass equal to that of younger individuals (Harber *et al*. 2012). When looking at muscle adaptive capacity to AE in ageing, attenuated vascular and muscular plasticity responses have been reported (Lawrenson *et al*. 2004) despite equal increases in maximal work rate and VO_2_ max after AE. However, at the whole‐body level, there is a decreased cardiovascular plasticity (Wang *et al*. 2014), indicating there may be attenuated responses to AE. Elderly individuals display reduced mitochondrial content in muscle (Conley *et al*. 2000), leading to the hypothesis that mitochondrial dysfunction may underlie blunted muscle oxidative capacity and the development of sarcopenia (Welle *et al*. 2003, Short *et al*. 2005a, Lanza *et al*. 2008, Liu *et al*. 2013). However, recently, Konopka *et al*. showed that, compared to younger counterparts, elderly individuals (~74 years) exhibit a comparable increase in MFN1, MFN2 and PGC‐1*α* protein content in skeletal muscle following 12 weeks aerobic exercise training (Konopka *et al*. 2014). Furthermore, AE training increases mitochondrial respiration, ATP production, enzyme activity and protein content to the same extent in younger (18–30 years) and older (59–76 years) individuals (Lanza *et al*. 2008), with lifelong training in older individuals retaining mitochondrial and PGC‐1*α* content such that older muscles are comparable to younger (Cobley *et al*. 2012). This suggests that irrespective of age and fitness, skeletal muscle still responds to exercise (and nutrition) to increase mitochondrial biogenesis. This adaptation appears to be most apparent in subsarcolemmal mitochondria, boosting electron transport chain enzyme activity (Menshikova *et al*. 2006). In addition to mitochondrial function, AE can also increase the protein content of the principal glucose transporter (GLUT4) and improve insulin action irrespective of age or gender (Cox *et al*. 1999). Beyond mitochondrial biogenesis, AE improves the muscle quality via increasing myosin heavy chain expression (Short *et al*. 2005b) and mixed MPS (Short *et al*. 2004). This is important because decrements in muscle function represent a major challenge in older age (Mitchell *et al*. 2012). As such, AE is an effective countermeasure to maintain skeletal muscle strength and functional capacity across the lifespan (Crane *et al*. 2013).

## Physiological and metabolic effect of disuse and ageing

Until now, the present review has focused upon the impact of exercise and nutrition in relation to the context of ageing and sarcopenia. As was discussed, exercise‐/nutrition‐mediated anabolism impact on muscle protein turnover (Atherton & Smith 2012) and thus muscle maintenance and adaptation to exercise, that is in healthy weight‐bearing humans, sufficient habitual physical activity and dietary protein intake are enough to ensure maintenance of skeletal muscle. However, when skeletal muscles are deprived of neural input through disuse (i.e. cast/crutch immobilization, bed rest) or sedentary lifestyles [a major behavioural concern impacting on modern culture (Oldridge 2008)], this can have devastating effects on the maintenance of skeletal muscle mass, metabolic health (Wall *et al*. 2013a) and physical functionality (De Boer *et al*. 2007a). This is precisely why both acute bouts of disuse or chronic sedentary behaviours are potentially so deleterious in the context of ageing. First, we must outline the basis of skeletal muscle atrophy. Decreases in muscle mass must ultimately be regulated through an imbalance between MPS and MPB. So which processes mechanistically regulate atrophy? Markers of MPB have shown to be upregulated early into disuse (Suetta *et al*. 2012), although it is generally believed that the majority of atrophy is due to attenuated MPS (Phillips *et al*. 2009) as the depression in MPS is calculably sufficient to explain muscle atrophy without the need for increases in MPB (De Boer *et al*. 2007b). We have shown that using static markers to infer changes in the dynamic processes of MPS and MPB is inherently flawed (Rennie *et al*. 2008) so any data using such markers should be interpreted with caution. Moreover, as the ‘atrogenes’, MAFBx and MuRF‐1 act to limit MPS by ‘tagging’ key initiation factors regulating the capacity for MPS, extra care should be taken in assigning their role exclusively to MPB (Koyama *et al*. 2008, Lagirand‐Cantaloube *et al*. 2008). Crucially, loss of muscle mass during disuse is a feature of both dysregulated post‐absorptive and postprandial MPS. For example, post‐absorptive MPS approximately halves after 10 days of lower limb suspension plateauing thereafter (De Boer *et al*. 2007b). This deficit is further enhanced by anabolic resistance in MPS in response to EAA stimuli (Glover *et al*. 2008b). Therefore, with MPB remaining relatively unchanged over 14 days of bed rest (Ferrando *et al*. 1996), increases in negative balance due to reduced post‐absorptive MPS and a reduced ability to reverse this negative balance during feeding cycles, due to blunted postprandial MPS, are likely to account for the prevailing loss in muscle CSA (De Boer *et al*. 2007b). However, there are suggestions that a transient and rapid increase in MPB during the initial first 3 days of immobilization may play an important role in this atrophy, but this is yet to be experimentally confirmed (Wall *et al*. 2013a), with the aforementioned ‘marker’‐based caveats mechanistic explanations for declines in MPS with immobilization are lacking. For example, associated proteins involved in anabolic cell signalling and translation initiation/elongation, such as mTOR, PKB, 4EBP1 and eEF2, remain unchanged during disuse adding complexity to the underlying mechanisms behind disuse atrophy (De Boer *et al*. 2007b, Wall *et al*. 2013b). FAK, a costameric localized and mechanosensing hub protein, is thought to play a key role in promoting MPS pathways (Crossland *et al*. 2013), is downregulated during disuse possibly explaining dampened MPS (De Boer *et al*. 2007b, Glover *et al*. 2008b). FAK has further shown to be involved in the load‐dependent remodelling of muscle (Li *et al*. 2013) and is upregulated after exercise (Wilkinson *et al*. 2008) and so along with other adhesome proteins, is likely to be key in disuse atrophy. Nonetheless, the mechanistic role of FAK remains unclear although it appears to be involved in the load‐induced hypertrophic response increasing protein translation through P70S6K (Klossner *et al*. 2009). Insight into the mechanisms behind attenuated MPS may be taken from animal studies in which, similar to that of human studies (De Boer *et al*. 2007b), limb immobilization in rats decreases MPS by 50% (Kelleher *et al*. 2013). Although unlike in humans, reduced MPS is linked with attenuated mTOR signalling. This appears to be induced by an upregulation of REDD1/2 (Kelleher *et al*. 2013) thought to be key regulators in the load‐induced activation of mTOR and MPS (Gordon *et al*. 2014). Further, immobilization results in an attenuated response to leucine, shown through reduced phosphorylation of P70S6K. Complete phosphorylation of P70S6K1 by mTOR requires phosphorylation by PDK1 and has recently been suggested to be proceed that of mTOR (Keshwani *et al*. 2011, Kelleher *et al*. 2013), yet the order in which these occur is still up for debate [discusses in Magnuson *et al*. (2012)]. However, these indicate PDK1 signalling plays a significant role in diminished MPS.

A key question remains: Do older people suffer more from the effects of disuse than younger people in terms of decline or rehabilitation? Without question, such rapid loss of muscle protein can present a serious problem for aged individuals; with many categorized as sarcopenic, accelerated muscle loss through short periods of bed rest may limit successful recovery, leading to the potential for reductions in overall physical activity and a vicious cycle of accelerated muscle loss and associated comorbidities. As with younger individuals, a decrease in muscle mass and resting MPS occurs rapidly with bed rest (Kortebein *et al*. 2007) with anabolic responses to EAAs being desensitized within 7 days (Drummond *et al*. 2012). Immobilization also yielded greater mass losses in younger vs. older individuals (Suetta *et al*. 2009, Hvid *et al*. 2010), although few studies have explored signalling differences with age (Suetta *et al*. 2012). Disuse atrophy is not limited to strict immobilization; periods of decreased activity by limiting step counts (something which is increasingly common with age) also significantly impact muscle health, decreasing insulin sensitivity and lipid metabolism in healthy young individuals (Olsen & Krogh‐Madsen 2008, Knudsen *et al*. 2012). When step count is limited in elderly persons, MPS shows a diminished response to EAAs, intensifying the effects of anabolic resistance (Breen *et al*. 2013). Crucially, compared to younger individuals, older people also appear to exhibit a lack of rehabilitative capacity in terms of regaining mass and function to pre‐bed rest values (Suetta *et al*. 2009, Hvid *et al*. 2010). On this basis, cumulative bouts of short‐term disuse, inactivity or chronic sedentary behaviours could contribute to the onset of impaired mobility and associated reductions in quality of life in ageing. On this basis, efforts need to be made to limit disuse‐associated muscle loss and to maximize recovery. In young subjects, EAA supplementation has shown little benefit in preventing muscle atrophy (Stein *et al*. 2003, Brooks *et al*. 2008), and aggressive EAA feeding with CHO has been shown to attenuate muscle loss, although this could be the result of attenuating decreased energy intake (Paddon‐Jones *et al*. 2004). Increasing protein intake to 1.6 g kg^−1^ body weight day^−1^ was unable to attenuate loss of muscle mass in older individuals during 5 days of bed rest (Dirks *et al*. 2014). Further, 45 g of additional EAAs provided in 3 doses per day increasing protein intake to 1.4 g kg^−1^ body weight day^−1^ in older individuals maintained basal MPS over a 24‐h period after 10 days of bed rest, despite failing to prevent muscle mass loss, again suggesting that the rapid increase in MPB and anabolic resistance to feeding may be a significant mechanism to this loss (Ferrando *et al*. 2010). More recent studies have shown that some nutritional supplements such as the leucine metabolite HMB, which is known to have potent affects on both MPS and MPB (Wilkinson *et al*. 2013), have potential to prevent or slow the decline in bed rest‐related muscle loss (Deutz *et al*. 2013), indicating the importance of nutritional strategies for assisting in combating the accelerated mass loss (Magne *et al*. 2013).

## Conclusions

Sensitization of aged muscle to stimuli central for muscle maintenance (nutrition and exercise) is impaired in older age. Despite a majority of data showing positive adaptations to exercise in youth and older age, these adaptive responses are vastly heterogeneous and appear (in the main but not totality) to be diminished in older age – perhaps due to suboptimal mechanical loading patterns and nutritional support. In some cases, it has been shown that age does not affect changes in strength and mass, although even individuals that continue to train into their 7th decade show decreases in muscle mass, strength and power (Faulkner *et al*. 2008, Mikkelsen *et al*. 2013), suggesting this is not an exclusively inactivity‐mediated phenomenon. Yet, exercise currently remains the most effective therapeutic strategy for countering sarcopenia (Frontera *et al*. 1988, Serra‐Rexach *et al*. 2011), and this may be associated with additional substantial health benefits, that is increases in strength, resting metabolic rate (Lemmer *et al*. 2001), glucose tolerance (Craig *et al*. 1989), reduced blood pressure (Hagberg *et al*. 1989) and improved lipid profile (Kelley *et al*. 2005). Finally, improved characterization of the influential behaviours (nutrition, physical activity, medications) of study volunteers could reduce contentious data as these can markedly affect study outcomes. It is hoped that the use of the novel tracer techniques, that is D_2_O (MacDonald *et al*. 2013, Wilkinson *et al*. 2014), D3 creatine (Clark *et al*. 2014) and D3 methylhistidine (Sheffield‐Moore *et al*. 2014) with more habitual application, will herald a new horizon for understanding the holistic (multi‐substrate) and longer‐term regulation of muscle metabolism in older age and to investigate the most effective means by which to promote healthy ageing.

## Conflict of interest

The authors report no conflicts of interest.

We would like to acknowledge support from the Medical Research Council UK and Arthritis Research UK (author D.J.W. is a MRC‐ARUK Centre of Excellence for Musculoskeletal Research into Ageing funded postdoctoral fellow (Grant: MRC MR/K00414X/1 and ARUK 19891)). [Correction added on 14 Aug 2015, after first online publication: The last paragraph was newly added]
